# Utilizing a novel digital affect mirror, morphii, to assess affective determinants of health

**DOI:** 10.1192/j.eurpsy.2021.354

**Published:** 2021-08-13

**Authors:** M. Milanak, B. Sullivan

**Affiliations:** 1 Psychiatry & Behavioral Sciences, Medical University of South Carolina (MUSC), Charleston, United States of America; 2 Research & Development, ADoH Scientific, Mt. Pleasant, United States of America

**Keywords:** Assessment, Affect, healthcare analytics, Whole person health

## Abstract

**Introduction:**

Decades of research have shown that affect, emotions and moods, significantly impact all aspects of health behaviors. This research utilized a novel digital analogue technology (Morphii) to assess eight affective domains: stress, anxiety, loneliness, irritability, depression, pain, energy and overall feelings of wellness.

**Objectives:**

To demonstrate the feasibility of use and strength of relationship/comparison to validated measures.

**Methods:**

A U.S. census-based sample of adults ages 18-80 (n=985) completed online assessments including the 8 Morphii’s and additional comparative mental/behavioral health assessments (PSS-4, GAD-7, UCLA Loneliness Scale V3, BITe, PHQ-8 & PHQ-2, P4 Pain Scale, WHO-5, CFQ-11, ESS, and Vitality Subscale SF-36) via the Prolific Academic online platform and were compensated nominally for their participation. The resulting sample was 51.6% female and 74.2% White.

**Results:**

Each Morphii was compared with the common corresponding industry assessment (e.g., Depression Morphii with PHQ) resulting in Pearson correlations ranging from -.519 to .761, with 6 of the 8 showing correlations above .700. Pearson correlations between dysfunction and each of the 8 Morphiis were significant at the p < .000 level, ranging from a low of .421 (Loneliness) to a high of .607 (Depression). Internal reliability was very good (Cronbach’s Alpha = .862). Respondents who expressed an assessment modality preference (55.2%) chose the Morphii type over traditional assessment format at a 2.5:1 ratio.

**Conclusions:**

Morphii provides a reliable and valid assessment option with the ability to obtain a comprehensive (8 domains at once), efficient (less than 60 second administration), assessment with increased patient/client preference and engagement.

**Disclosure:**

Milanak - submitting author - I serve on the advisory board for ADoH Scientific to consult on scientific research of Morphii development. To date, I have not been paid any money for this advisory role.

